# Early Cardiac Allograft Vasculopathy: Are the Viruses to Blame?

**DOI:** 10.1155/2012/734074

**Published:** 2012-05-31

**Authors:** Ashim Aggarwal, Joseph Pyle, John Hamilton, Geetha Bhat

**Affiliations:** ^1^Center for Heart Transplant and Assist Devices, Advocate Christ Medical Center, Oak Lawn, IL 60453, USA; ^2^Department of Pathology, Advocate Christ Medical Center, Oak Lawn, IL 60453, USA

## Abstract

This paper describes a case of early (7 months after transplant) cardiac allograft vasculopathy. This-43-year-old (CMV positive, EBV negative) female patient underwent an orthotopic heart transplant with a (CMV negative, EBV positive) donor heart. She had a history of herpes zoster infection and postherpetic neuralgia in the past. The patient's panel reactive antibodies had been almost undetectable on routine surveillance testing, and her surveillance endomyocardial biopsies apart from a few episodes of mild-to-moderate acute cellular rejection (treated adequately with steroids) never showed any evidence of humoral rejection. The postoperative course was complicated by multiple admissions for upper respiratory symptoms, and the patient tested positive for entero, rhino, and coronaviruses serologies. During her last admission (seven months postoperatively) the patient developed mild left ventricular dysfunction with an ejection fraction of 40%. The patient's endomyocardial biopsy done at that time revealed concentric intimal proliferation and inflammation resulting in near-total luminal occlusion in the epicardial and the intramyocardial coronary vessels, suggestive of graft vasculopathy with no evidence of rejection, and the patient had a fatal ventricular arrhythmia.

## 1. Introduction

Cardiac transplantation is a well-defined therapy for end-stage heart failure. Following transplantation, median survival is 10 years rising to 13 years for those who survive the first year [1]. The leading causes of death in the first year following transplantation include infection, rejection, and graft failure. Cardiac allograft vasculopathy (CAV) is the second leading cause of death after 1 year following transplantation, second only to malignancy [1].

We present a case of early (7 months after transplant) cardiac allograft vasculopathy (CAV) in a cytomegalovirus (CMV) positive patient with a history of herpes zoster infection and multiple other viral infections in the postoperative course possibly contributing to graft vasculopathy.

## 2. Case

A 43-year-old Caucasian female with a history of nonischemic dilated cardiomyopathy with left ventricular ejection fraction (LVEF) of 10–15% status following Thoratec Heart-Mate II left ventricular assist device (LVAD) (implanted 2 years prior as a Bridge to Transplant) was transferred to our tertiary care facility for management of unresolving pseudomonas driveline infection. The patient secondary to persistent pseudomonas bacteremia despite adequate treatment with intravenous antibiotics underwent LVAD removal with reimplantation with another VAD. The patient also underwent an AICD lead extraction with generator change secondary to questionable vegetation on the defibrillator lead on transesophageal echocardiogram. The patient did well following that and remained home for 4 months while awaiting a cardiac transplant. Her past history was significant for hypertension, dyslipidemia, recurrent pulmonary embolism, history of herpes zoster infection with postherpetic neuralgia, and intracerebral hemorrhage.

Four months later the patient was electively admitted for transplant evaluation. Her panel reactive antibody (PRA) levels were found to be low at 4% as measured by flow cytometry using HLA class I Luminex-coated beads. The patient (CMV positive) finally underwent a CMV negative, Epstein-Barr virus (EBV) positive orthotopic heart transplant without the need for desensitization. The patient's immediate postoperative course was complicated by multiple failed attempts at extubation secondary to fluid overload that required tracheostomy and acute kidney injury requiring temporary hemodialysis (with complete eventual recovery of renal function). The patient after 4 weeks, on routine surveillance endomyocardial biopsy (EMB), was found to have ISHLT grade 2R acute cellular rejection which was successfully treated with intravenous pulsed steroids and mycophenolate mofetil. The patient was eventually discharged home 2 weeks later and was followed as an outpatient. Three months subsequent to transplant the patient started to develop signs and symptoms of upper respiratory tract infections manifesting as unremitting cough. The patient admitted was found to have viral infection with positive serologies for entero, rhino, and coronaviruses, and the EMB was negative for rejection. The patient was managed conservatively without any antiviral treatment except prophylactic ganciclovir for CMV prophylaxis and discharged home. The patient did present again with similar respiratory symptoms a month later at which time it was decided to treat the patient with a course of oseltamivir (Tamiflu) for a clinical suspicion of influenza. The patient was discharged only to be readmitted 2 months later (6 months after transplant) for symptoms of exertional dyspnea, nausea, and abdominal pain. The patient was found to have low cardiac index (1.59 L/min/m^2^) and elevated right sided pressures on right heart catheterization while the EMB remained negative for cellular or humoral rejection. An echocardiogram at the time revealed a mildly depressed left ventricular ejection fraction at 40% with mild right ventricular dysfunction. The patient's panel reactive antibodies were undetectable.  Table 1 lists the trends in the available viral titres and other laboratory data (glucose and lipids). The patient was treated with intravenous methylprednisolone and plasmapheresis to treat for possible graft dysfunction. The next day the patient had a sudden cardiorespiratory arrest and died despite prolonged attempts at resuscitation.

**Figure 1:**
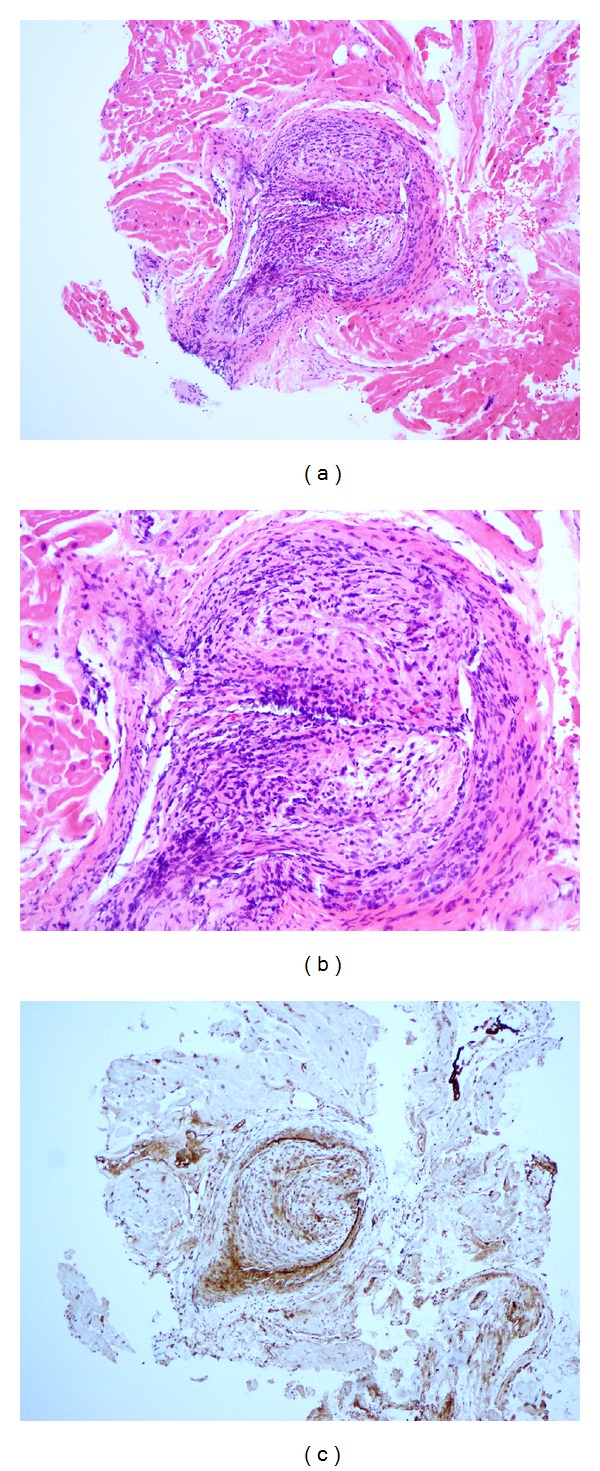
Endomyocardial biopsy 7 months after transplant showing graft vasculopathy. Endomyocardial biopsy demonstrating microscopic changes of concentric intimal proliferation and chronic inflammation resulting in near-total luminal occlusion in the epicardial and the intramyocardial coronary vessels, suggestive of graft vasculopathy. Hematoxylin and eosin stain: (a) low power, (b) high power, and (c) C4d immunostaining. Immunoperoxidase with C4d monoclonal antibody negative.

A postmortem analysis revealed microscopic changes of concentric intimal proliferation and inflammation resulting in near-total luminal occlusion in the epicardial and the intramyocardial coronary vessels, suggestive of graft vasculopathy (Figures 1(a)–1(c)). There was no evidence of rejection seen.

## 3. Discussion

Cardiac transplantation is the definitive treatment for eligible patients with end-stage heart failure. CAV and graft failure are the leading cause of death in patients who survive the first year after transplant. Despite a small decrease in the cumulative incidence documented recently, the incidence of CAV after transplant remains significant: 8% at 1 year, 20% at 3 years, 30% at 5 years, and more than 50% at 10 years [1]. CAV is a risk factor for long-term mortality, but the diagnosis of CAV also carries a short-term mortality risk—approximately 10% of patients die in the 12 months after the diagnosis CAV [1].

The diagnosis of CAV is challenging because of deinnervation as well as the concentric and diffuse nature of the disease. Classical symptoms of angina are often missing, and patients tend to present with heart failure [2]. There are multiple purported etiologies for graft coronary artery disease including both immunologic and nonimmunologic factors. Immunologic factors include human leukocyte antigen mismatching, cytokine production, and activation of the cellular immune system. Nonimmunologic factors include diabetes, hypertension, and hyperlipidemia [3, 4]. There is also accumulating data suggesting that infections play a role in atherosclerosis and in CAV.

CMV is one of the pathogens that has been most convincingly implicated in the pathophysiology of CAV [5]. Viral infections including those due to CMV have been associated with accelerated CAV [6, 7]. Positive pretransplantation CMV serology has been shown to be a risk factor for CAV in children [8, 9]. Cardiovascular risk has also been associated with seropositivity for Chlamydia pneumonia, Helicobacter pylori, CMV, and other herpes viruses. However, more recent studies in larger patient cohorts have demonstrated only modest associations [10, 11]. Given the modest and variable risk that has been linked to individual pathogens, Zhu and colleagues have proposed the sum of the relevant infectious agents, defined as the “total pathogen burden,” as the important risk factor to be considered. Exposure to a panel of five pathogens was found to be predictive of angiographic disease [12] and cardiovascular events [13]. Similar results were reported in a study showing that exposure to increasing number of pathogens was associated with increased risk [14]. An increasing “pathogen burden” defined as the aggregate number of positive serologies (mainly driven by burden of Herpesviridae) was significantly predictive of the long-term prognosis in a doseresponse manner. These observations have led the investigators to propose the “Herpes Burden” (aggregate seropositivity to CMV, herpes simplex-1 and -2, and EBV) as a more effective predictor of cardiovascular risk [14]. Our patient had a CMV positive status who received an EBV positive donor heart along with a history of documented herpes zoster infection in the past. Whether the multiple serologically positive viral infections (entero, rhino, and coronaviruses) amongst the many other nonimmune factors augmented the existent viral burden thereby triggering a self-perpetuating immune cascade, inducing inflammation and intimal proliferation, is a concept that can only be hypothesized and arduous to prove. Kumar et al. in their prospective study of 93 lung transplant recipients recently demonstrated acute rejection (≥grade 2 ISHLT) in 33.3% (16/48) patients within 3 months of respiratory viral infections (RVIs) as opposed to only 6.7% (3/45) in RVI negative patients. Sixty-two percent of these patients with history of RVIs progressed to obliterative bronchiolitis (chronic airway rejection) on biopsies done within 1 year [15]. This study adds to the increasing evidence highlighting a possible immunogenic potential of respiratory viruses in solid organ recipients manifesting as acute or chronic rejection.

Treatment of established CAV has been disappointing. The options have included adjusting the immunosuppressive regimen, percutaneous or surgical revascularization or retransplantation. Because CAV has such an important influence on morbidity and mortality, it is evident that if further improvement in graft and patient survival is to be made, attention must focus on reducing the risk of CAV. There is proven benefit from lipid lowering therapy, mammalian target of rapamycin (mTOR) inhibitors (everolimus and sirolimus), and possibly diltiazem (calcium channel blocker) [4].

Our case is an addition to a list of very limited number of cases of early allograft vasculopathy (within the first year after transplant) that aims to highlight its poor clinical outcome and survival compared to the form that develops after the first year. The lack of data on the patients viral titres is an important limitation of this case. Allograft coronary disease remains one of the greatest obstacles to long-term recipient survival after cardiac transplantation. This process has been extensively studied, yet remains poorly understood. Although it typically becomes clinically significant years after transplantation, it can also occur early and result in recipient death as early as 6 months following transplant. Why a given patient develops an aggressive form of the disease early after transplantation and another patient may live ten or even twenty years without evidence of clinically significant disease is unknown.

## Figures and Tables

**Table 1:** Viral titres and lab data before and after transplant.

	Pretransplant	3 months after	6 month after
		transplant	transplant
CMV IgG	3.56	NA	Undetectable
EBV IgG	>1.09	NA	NA
VZV IgG	5.04	NA	NA
Glucose (mg/dL)	122	154	150
Total cholesterol (mg/dL)	185	200	190
LDL-C (mg/dL)	123	125	120
HDL-C (mg/dL)	32	30	32
TG (mg/dL)	149	168	160

CMV: cytomegalovirus, EBV: Epstein-Barr virus, HDL: high-density lipoprotein, IgG: immunoglobulin G, NA: data not available, LDL: low-density lipoprotein, TG: triglyceride, VZV: varicella zoster virus (normal values for all viral titres: <0.90 in our laboratory).
